# The Impact of Metabolic Syndrome on the Outcomes of Rehabilitation in Post-COVID-19 Patients

**DOI:** 10.3390/jcm14165893

**Published:** 2025-08-21

**Authors:** Alicja Mińko, Agnieszka Turoń-Skrzypińska, Aleksandra Rył, Iwona Rotter

**Affiliations:** Department of Medical Rehabilitation and Clinical Physiotherapy, Pomeranian Medical University, 71-210 Szczecin, Poland; agi.skrzypinska@gmail.com (A.T.-S.); aleksandra.ryl@pum.edu.pl (A.R.); iwrot@wp.pl (I.R.)

**Keywords:** recovery of function, SARS-CoV-2 infection, syndrome X

## Abstract

**Background/Objectives**: The coexistence of metabolic syndrome and COVID-19 presents a major challenge for healthcare systems, as individuals with metabolic syndrome are at significantly higher risk of severe disease and poor outcomes. The aim of this study was to assess how metabolic syndrome influences the outcomes of rehabilitation in patients recovering from COVID-19. **Methods**: This is a prospective observational study conducted at a rehabilitation hospital in Szczecin (Poland). One hundred and forty-six patients with COVID-19 were enrolled. Data on age, gender, BMI, comorbidities, and hospitalization were analyzed. The intervention included a comprehensive post-COVID-19 rehabilitation program. Data were collected using functional tests (6MWT and spirometry), and clinical records were analyzed. **Results**: Patients with metabolic syndrome had significantly higher BMI (*p* < 0.001), lower HDL cholesterol (*p* < 0.001), and higher triglyceride levels (*p* < 0.001) compared with the group without metabolic syndrome. After rehabilitation, both groups showed significant improvements in 6MWT distance (with MetS *p* < 0.001; without MetS *p* < 0.001) and FEV_1_% predicted (MetS *p* = 0.025; without MetS *p* = 0.021). However, regression analysis shows that age was a significant negative predictor of 6MWT performance in both groups (*p* < 0.01), whereas hypertension and diabetes predicted worse post-rehabilitation outcomes in the group without MetS. **Conclusions**: Comprehensive rehabilitation after COVID-19 benefits all patients, regardless of the presence of metabolic syndrome. However, individual clinical factors such as age, hypertension, diabetes, and male gender are crucial to its effectiveness. This highlights the need for individualized rehabilitation programs, especially for patients with metabolic conditions, which can significantly increase the effectiveness of therapeutic interventions.

## 1. Introduction

The co-occurrence of two pandemics—metabolic syndrome and COVID-19—over recent years has posed a significant challenge to healthcare systems worldwide. Evidence suggests a markedly increased risk of adverse COVID-19 outcomes in individuals with metabolic syndrome. However, little is known about differences in treatment and recovery from SARS-CoV-2 infection between individuals with and without metabolic syndrome [1,2].

Metabolic syndrome refers to the clustering of several risk factors, such as overweight or obesity, hypertension, dyslipidemia, and insulin resistance, which collectively increase the risk of cardiovascular disease. Its prevalence is rising globally, with estimates suggesting that it affects approximately 25% of the population [1,3]. Individuals with metabolic syndrome are at greater risk of poor prognosis and mortality from COVID-19. Studies indicate that metabolic syndrome is associated with a 2.3-fold higher risk of COVID-19-related death. Previous reports have shown that obesity, diabetes, and hypertension significantly increase the likelihood of severe COVID-19, including hospitalization, admission to intensive care units, and death [1,4].

Metabolic syndrome and COVID-19 demonstrate strong pathophysiological links, with a common denominator being chronic inflammation and dysregulation of the immunometabolic response. Individuals with metabolic syndrome exhibit elevated levels of proinflammatory cytokines, such as IL-6, TNF-α, and CRP, which contribute to the cytokine storm in COVID-19 and may lead to organ damage. Furthermore, insulin resistance and hyperglycemia impair the immune system, limiting the ability to effectively eliminate the virus. The ACE2 receptor, the primary entry point for SARS-CoV-2, is overexpressed in adipose tissue, which may increase the viral reservoir and exacerbate the infection in individuals with obesity [5,6,7,8]. Obesity is also associated with other physiological alterations that may worsen the clinical course of COVID-19, including increased ventilatory demand, elevated respiratory workload, respiratory muscle insufficiency, and impaired pulmonary ventilation leading to oxygen desaturation. Patients with metabolic syndrome require close monitoring due to the elevated risk of disease progression [6,7]. These molecular mechanisms may explain why patients with metabolic disorders are more susceptible to severe COVID-19 and have longer recovery after infection.

Given the long-term complications of COVID-19—especially in individuals with pre-existing conditions—a multidisciplinary approach is essential. Post-COVID-19 complications can persist for an extended period, often resulting in multifactorial disability. The most common disorders include dyspnea, chronic fatigue, weakness, muscle pain, joint mobility limitations, myopathy, and impairments in balance, stability, and coordination. Additionally, these patients are at high risk of developing mental health disorders and post-intensive care syndrome (PICS) [9,10,11].

To improve functional outcomes in post-COVID-19 patients, physiotherapy should be an integral part of the treatment process. Substantial evidence supports the effectiveness of post-COVID-19 rehabilitation in enhancing physical performance, muscle strength, coordination, and mental health status [12,13,14]. Despite clear evidence of the increased risk of poor COVID-19 outcomes in individuals with metabolic syndrome, differences in the efficacy of physiotherapeutic interventions in this population have received limited attention in the literature.

Despite increasing awareness of the long-term consequences of SARS-CoV-2 infection, the impact of pre-existing metabolic disorders on the outcomes of post-COVID-19 rehabilitation remains insufficiently understood. Although metabolic syndrome is a well-established risk factor for severe COVID-19, there is a lack of comprehensive data on how it may affect recovery trajectories and rehabilitation efficacy in this population.

The present study addresses this knowledge gap by hypothesizing that post-COVID-19 patients with metabolic syndrome achieve less favorable functional outcomes following rehabilitation than those without the syndrome. The rationale for this hypothesis lies in the pathophysiological interactions between metabolic syndrome and COVID-19, including chronic systemic inflammation, immune dysregulation, and impaired pulmonary and muscular function.

The aim of this study was to assess how metabolic syndrome influences the outcomes of rehabilitation in patients recovering from COVID-19.

## 2. Materials and Methods

### 2.1. Study Setting and Patient Enrollment

This study is a prospective observational study. Patient recruitment for the study took place from May 2021 to September 2022. Recruitment took place at the St. Charles Borromeo Rehabilitation Hospital in Szczecin, where patients were undergoing inpatient therapeutic rehabilitation following SARS-CoV-2 infection. The Post-COVID-19 Rehabilitation Unit admitted individuals recovering from COVID-19 based on a referral from their family physician. Final qualification for post-COVID-19 rehabilitation was performed by a medical rehabilitation physician from the rehabilitation hospital, applying the criteria specified in the National Health Fund (NFZ) guidelines, in accordance with Order No. 42/2021/DSOZ of the President of the NFZ of 5 March 2021 [15].

Qualification for the Post-COVID-19 Rehabilitation Unit required the presence of post-COVID-19 complications, assessed according to the following criteria:

- Post-COVID-19 Functional Status (PCFS) Scale (score 1–4);

- Medical Research Council (MRC) scale (score < 5);

- Modified Medical Research Council (mMRC) dyspnea scale (score ≥ 1).

The Post-COVID-19 Functional Status Scale is a five-point tool used to assess functional limitations across various health domains after SARS-CoV-2 infection [16]. The Medical Research Council scale evaluates muscle strength on a scale from 0 to 5, where 0 indicates no muscle contraction, and 5 represents normal strength [17]. The modified Medical Research Council scale measures the severity of dyspnea, ranging from 0 (dyspnea only during strenuous activity) to 4 (dyspnea preventing the patient from leaving home) [18].

A total of 171 patients were initially screened. After applying all inclusion and exclusion criteria, 146 patients were included in the final analysis. Participants eligible for inclusion were adults (aged 18 and over) with a confirmed SARS-CoV-2 infection, verified by a positive PCR test. To qualify, individuals had to have completed COVID-19 treatment within the preceding 12 months and provide informed consent. Exclusion criteria included any condition that impaired the ability to give informed consent or understand the study’s purpose and procedures. Additional exclusions applied to those with neurodegenerative or neurodevelopmental conditions, musculoskeletal disorders limiting participation in rehabilitation, or severe cardiovascular disease. Individuals diagnosed with epilepsy, malignancies, or those with contraindications to spirometry or the 6 min walk test (6MWT) were also excluded.

Patients were divided into two groups: those with metabolic syndrome and those without. The classification was based on established diagnostic criteria for metabolic syndrome, which include the following:

- Waist circumference ≥ 102 cm in men or ≥88 cm in women, or a body mass index (BMI) ≥ 30 kg/m^2^, and the presence of at least two of the following three criteria:

- Fasting plasma glucose > 100 mg/dL or 140 mg/dL at 120 min during oral glucose tolerance testing, or HbA1c ≥ 5.7%, or treatment with oral hypoglycemic agents.

- Elevated non-HDL cholesterol (≥130 mg/dL) or ongoing lipid-lowering therapy.

- Systolic blood pressure > 130 mmHg and/or diastolic blood pressure > 85 mmHg, or pharmacological treatment of previously diagnosed hypertension [19].

Each patient provided written informed consent to participate in the study and for the use of their medical records. Patient privacy and anonymity were maintained throughout. The study was conducted in compliance with the current version of the Declaration of Helsinki and was approved by the Bioethics Committee of the Pomeranian Medical University in Szczecin (approval No. KB-0012/15/2021; date: 31 May 2021). In addition, the second approval is an extension of the first (protocol code KB-0012/15/2021-A-42 of 28 June 2023).

The study was conducted in accordance with STROBE (Strengthening the Reporting of Observational Studies in Epidemiology) guidelines [20].

### 2.2. Study Process

Patients participated in an inpatient rehabilitation program. The full course of rehabilitation lasted a minimum of 2 weeks and a maximum of 6 weeks. The duration was determined by the attending physician based on a comparison of clinical assessments performed before and after the rehabilitation program.

Rehabilitation sessions were conducted six times per week and included the following:

- Breathing exercise (active breathing exercises, active breathing exercises with resistance, and learning effective coughing and clearing of the airways; duration: 30 min per day).

- Aerobic training (stair climbing, outdoor walking, continuous/interval training on a cycle ergometer, exercise tolerance assessed by oxygen saturation (via pulse oximeter) and perceived exertion using the Borg scale; gradual increase in intensity by 5–10%; duration: 90 min per day).

- Strength and endurance training (training individually tailored to the patient based on one repetition maximum (1 RM) and exercise tolerance (assessed via desaturation); load: 70–85% of 1 RM; volume: 3 sets of 8–12 repetitions; rest: 1–2 min between sets; progression starting at 60–70% of 1 RM; duration: 30 min per day).

Throughout the entire rehabilitation period, patients remained under continuous medical, nursing, and physiotherapeutic supervision.

Anthropometric measurements, such as body weight and height, were taken from the patients’ medical records (measurements were taken by medical staff on the day of admission to the rehabilitation unit). Waist circumference was assessed using a flexible, non-stretchable measuring tape for medical use (ADE MZ10021, ADE Germany GmbH, Hamburg, Germany).

Pulmonary function was assessed using spirometry with the BTL-08 Spiro device (BTL Industries Ltd., Newcastle-under-Lyme, UK). The following parameters were measured: forced expiratory volume in the first second (FEV_1_), forced vital capacity (FVC), and the FEV_1_/FVC ratio. FEV_1_ and FVC values were expressed as percentages of predicted normal values automatically calculated based on the patient’s age, sex, height, weight, and ethnicity. The FEV_1_/FVC ratio was reported as an absolute value. The spirometry results were interpreted according to the ECCS/ERS 1993 reference values [21]. All spirometric measurements were conducted following the standard guidelines of the American Thoracic Society (ATS) and the European Respiratory Society (ERS) [22].

Exercise capacity was evaluated using the 6MWT, performed according to ATS and ERS standards [23]. The results were expressed both as absolute distance (in meters) and as a percentage of predicted normal values. Predicted values were calculated using the following equations:

- For men: 6MWT distance (m) = (7.57 × height [cm]) − (5.02 × age [years]) − (1.76 × weight [kg]) − 309
- For women: 6MWT distance (m) = (2.11 × height [cm]) − (2.29 × weight [kg]) − (5.78 × age [years]) + 667

Based on the measured distances and the formula speed = distance/time (m/s), the average walking speed was calculated.

All assessments were performed twice: on the first day of rehabilitation (Measurement 1) and on the final day of the rehabilitation program (Measurement 2).

On the first day of rehabilitation, venous blood samples were collected from the antecubital vein to evaluate the lipid profile, including total cholesterol, HDL cholesterol, LDL cholesterol, and triglycerides. Blood collection was performed by trained nurses in accordance with standard protocols. Blood was drawn into tubes containing ethylenediaminetetraacetic acid (BD Vacutainer^®^, Becton Dickinson, NJ, USA). Plasma was separated by centrifugation, frozen, and stored at −80 °C until laboratory analysis.

Sociodemographic data were also obtained through a structured interview conducted on the first day of rehabilitation. Detailed information regarding the course of COVID-19, treatment received, and comorbidities was obtained from an analysis of the patients’ medical records.

**Figure 1:**
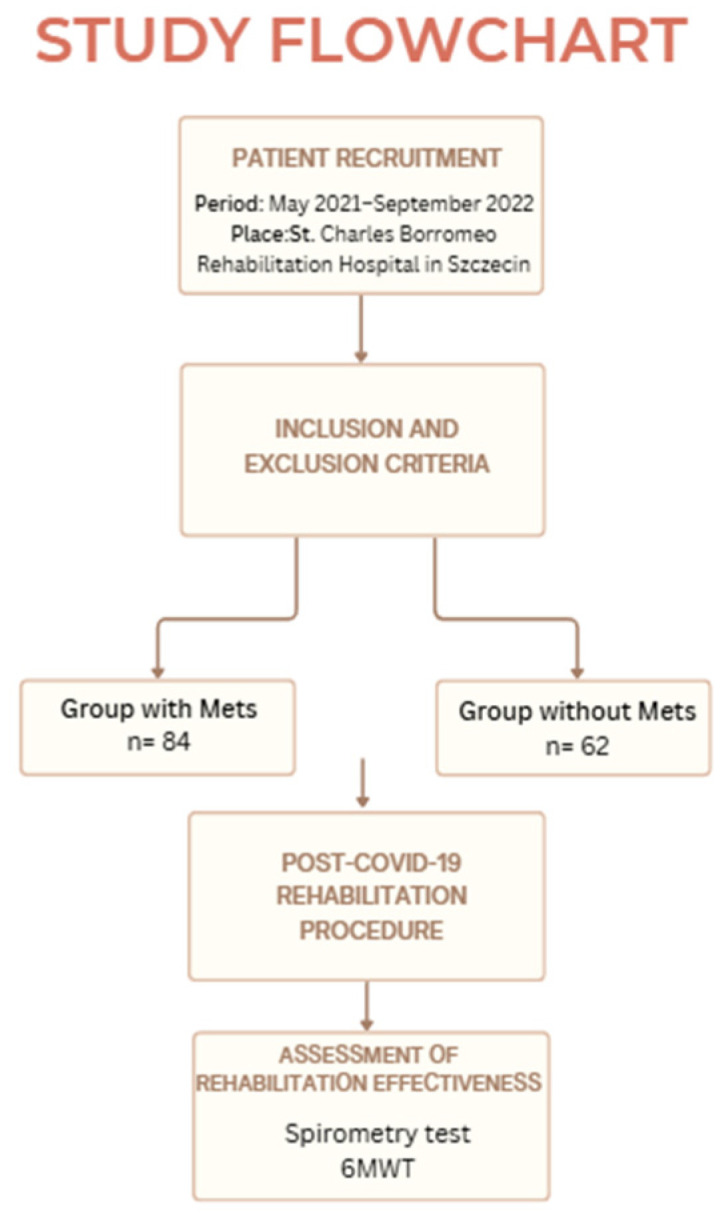
A flowchart of the study.

A simplified flowchart of the study is presented in Figure 1.

### 2.3. Statistical Analyses

Statistical analysis was performed using Statistica 13.1 software (StatSoft, Inc., Tulsa, OK, USA). Characterization of the study group was performed, taking into account the number of patients, patients’ percentage, mean, median, first and third quartiles, and standard deviation. Normality of distribution was tested using the Shapiro–Wilk test. For the normal distribution, the data are presented in the mean and standard deviation. For the non-normal distribution, the data are presented in the median, first, and third quartiles. The differences between the two groups were analyzed using Student’s *t*-test and the Mann–Whitney U-test. Nominal variables were tested using the chi-squared test. Dependent variables were tested with the *t*-test for dependent samples and the Wilcoxon test. Multivariable logistic regression analysis was performed. The model for multivariable logistic regression was adjusted for gender, age, diabetes, and hypertension. Bonferroni correction was applied for multiple comparisons. An a priori sample size calculation was not performed during the planning phase of the study. To transparently address this limitation, a post-hoc power analysis was conducted after data collection to estimate the minimum required number of participants needed to detect the observed effects. The analysis was performed using a two-sample *t*-test, comparing the mean improvement in the 6MWT between the metabolic syndrome (MetS) and non-MetS groups. Based on these calculations, it was determined that to detect the observed effect (a small effect size, Cohen’s d = 0.24) with 80% statistical power and a significance level of 0.05, the minimum required sample size in each group should have been 278 patients. A *p*-value of <0.05 was regarded as statistically significant.

## 3. Results

Table 1 presents the characteristics of the study population, stratified by the presence of metabolic syndrome. A total of 84 patients (57.5%) were diagnosed with metabolic syndrome, while 62 patients (42.5%) did not meet the diagnostic criteria.

Comparative analysis reveals significant differences in several clinical variables. Patients with metabolic syndrome had significantly higher BMI values (*p* < 0.001) and were more likely to have developed pneumonia during the course of COVID-19 (*p* = 0.004). Comorbid conditions such as diabetes mellitus (*p* < 0.001) and hypertension (*p* < 0.001) were also significantly more common in this group.

Table 2 presents a comparison of lipid profile parameters between patients with metabolic syndrome and those without. HDL cholesterol levels were significantly lower in the metabolic syndrome group compared to the non-MetS group (*p* < 0.001). Triglyceride (TG) levels were significantly higher in the MetS group than in the non-MetS group (*p* < 0.001).

Table 3 presents the results of multivariate regression analysis for the MetS group. In the MetS group, age was found to be the only statistically significant factor influencing exercise capacity measured by the 6MWT. Analysis shows that older age was significantly associated with poorer performance on this test, both at the beginning (*p* = 0.002) and at the end of rehabilitation (*p* = 0.002).

Table 4 presents the results of multivariate regression analysis for the group without metabolic syndrome. In the group without metabolic syndrome, age predicted the 6MWT results both at baseline (*p* < 0.001) and at the end of rehabilitation (*p* < 0.001). Hypertension was a factor influencing 6MWT distance after rehabilitation (*p* = 0.011). Diabetes was a significant factor influencing FVC results at the final post-rehabilitation measurement (*p* = 0.024). Male gender predicted FVC results at the final post-rehabilitation measurement (*p* = 0.038).

**Figure 2:**
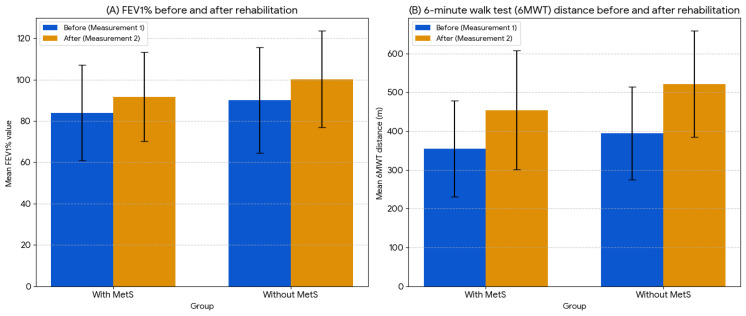
Comparison of mean FEV1% and 6MWT distance values before and after rehabilitation.

Figure 2 compares the mean FEV1% and 6MWT before and after rehabilitation within the pre- and post-rehabilitation groups. Improvements in FEV1 and the 6MWT were observed in both patients with and without metabolic syndrome. Patients with MetS achieved significant improvements in the 6MWT (*p* < 0.001) and FEV1 (*p* = 0.025). Patients without MetS also achieved significant improvements in the 6MWT (*p* < 0.001) and FEV1 (*p* = 0.021).

## 4. Discussion

The present study aimed to evaluate the impact of metabolic syndrome on the outcomes of a comprehensive post-COVID-19 rehabilitation program. The central research question was whether individuals with metabolic syndrome experience similar functional benefits from rehabilitation as those without the syndrome. Based on the existing literature linking metabolic syndrome with impaired pulmonary function and reduced exercise tolerance, we hypothesized that patients with metabolic syndrome would demonstrate smaller improvements in spirometry and 6MWT parameters following rehabilitation.

Coronavirus disease (COVID-19) has been shown to negatively affect respiratory function and physical performance, even in the months following recovery. Patients often experience reductions in spirometric parameters such as FVC and FEV1, as well as decreased 6 min walk test (6MWT) distance, particularly among those who required hospitalization or oxygen therapy during the acute phase of infection [24,25].

In parallel, metabolic syndrome (MetS)—characterized by abdominal obesity, dyslipidemia, hypertension, and insulin resistance—is independently associated with impaired lung function and reduced exercise tolerance. MetS contributes to low-grade systemic inflammation, endothelial dysfunction, and muscular deconditioning, all of which may interfere with rehabilitation outcomes. The combined burden of COVID-19 and metabolic syndrome may have an additive, or even synergistic, negative impact on physical function, likely mediated by overlapping inflammatory and metabolic [4,26,27,28].

However, our results confirm that this relationship may not be strictly proportional. Our findings confirm that comprehensive rehabilitation after COVID-19 is beneficial for all patients, regardless of the presence of metabolic syndrome. However, we demonstrate that attributing poorer outcomes solely to metabolic syndrome is problematic. After adjusting for age, gender, diabetes, and hypertension, metabolic syndrome alone was no longer a statistically significant predictor of poorer rehabilitation outcomes. We reveal that individual factors, not the overall syndrome, play a key role in the recovery process. Age emerged as a consistent predictor of diminished functional recovery in 6MWT performance. Interestingly, in the non-MetS group, hypertension and diabetes were significant negative predictors of rehabilitation response, suggesting that even outside the full MetS phenotype, specific metabolic comorbidities can independently influence recovery trajectories.

These observations align closely with the findings of a recent meta-analysis by Li et al. [29], which reported that while structured pulmonary rehabilitation offers clear benefits for lung function and exercise capacity in patients with long COVID, the magnitude of these improvements is attenuated by the presence of comorbid conditions, particularly those of metabolic origin. This reinforces the notion that a “one-size-fits-all” approach to rehabilitation may be insufficient, especially in complex patient populations with overlapping physiological impairments.

Our regression analysis reveals that age was the most consistent independent predictor of lower exercise capacity (6MWT) both before and after rehabilitation in both MetS and non-MetS patients, which is in line with previous findings linking aging to reduced cardiorespiratory fitness and slower functional recovery in chronic disease populations [25,30]. In the non-MetS group, hypertension and diabetes emerged as additional negative predictors, suggesting that specific cardiometabolic components may independently affect rehabilitation response even in the absence of full MetS criteria.

These findings align with the meta-analysis by Li et al. [29], which demonstrated that while pulmonary rehabilitation improves lung function and exercise tolerance in long COVID, the magnitude of improvement is attenuated by comorbidities, especially metabolic disorders. Similarly, Sonnweber et al. [31] reported delayed pulmonary recovery in post-COVID-19 patients with pre-existing metabolic diseases, emphasizing the additive burden of systemic inflammation and metabolic dysregulation.

Comprehensive rehabilitation led to significant improvements in both study groups of post-COVID-19 patients. The literature consistently highlights the effectiveness of rehabilitation in improving exercise capacity and pulmonary function in post-COVID-19 patients. Numerous studies have shown that pulmonary rehabilitation programs incorporating aerobic and resistance training enhance lung function and exercise tolerance—as measured by 6MWT distance—in individuals recovering from COVID-19 [29,32,33,34].

Previous studies indicate that the presence of metabolic factors, such as obesity, hypertension, type 2 diabetes, or dyslipidemia, does not preclude achieving positive outcomes from physiotherapy based on physical training. Although individuals with metabolic syndrome typically begin rehabilitation with lower physical fitness levels and reduced exercise tolerance, they demonstrate marked improvement in functional and clinical parameters in response to appropriately tailored aerobic and resistance training. While the response to exercise interventions in this group may be slower, multicenter studies show that even a moderate increase in physical activity leads to improvements in 6MWT distance and spirometric values [30,35,36].

Our findings suggest that while standard rehabilitation programs benefit all patients, they may be insufficient to completely eliminate performance differences among older individuals or those with underlying conditions such as diabetes or hypertension.

These findings have important implications for clinical practice. The data presented support the need to adapt standard rehabilitation programs to better meet the needs of older patients and those with metabolic disorders.

In summary, our study highlights the importance of individualized rehabilitation strategies for post-COVID-19 patients, taking into account comorbidities. Although standard rehabilitation protocols are beneficial, they may require adaptation to fully meet the needs of patients with metabolic disorders. Further research is needed to determine optimal intervention parameters and assess long-term outcomes.

### Limitations

One limitation of our study was the variability in the time from recovery to the initiation of rehabilitation; however, none of the patients began rehabilitation more than 12 months after recovering from COVID-19. The duration of the rehabilitation cycle was also not uniform, ranging from 2 to 6 weeks, which may have influenced the final outcomes. Another limitation of the study is the lack of detailed information regarding the participants’ acute COVID-19 treatment. Differences in therapeutic methods used during the acute phase of the disease may have influenced the recovery process. Another limitation is that the sample size was estimated at 278, but the actual sample size was 146. This small sample size may have been too small to detect some statistically significant differences, despite their clinical significance. Future studies should consider increasing the number of participants. A limitation of the study is the lack of data on the functional status of patients before COVID-19, which may have influenced their response to rehabilitation. Future studies should consider including this information. Another limitation of this study is the lack of long-term follow-up, which prevents assessment of the durability of the achieved therapeutic effects and the risk of relapse. Future studies recommend conducting long-term analyses to verify the maintenance of rehabilitation effects and assess the need for follow-up interventions. Another limitation of this study is the lack of available data from chest imaging (CT and X-ray) and functional respiratory tests (DLCO and CPET), which could help differentiate the effects of metabolic syndrome from potential permanent pulmonary changes after COVID-19. Future studies are recommended to incorporate a complex multimodal assessment combining imaging and functional methods.

A key strength of the study was the supervised nature of the rehabilitation process: all patients performed exercises within a dedicated rehabilitation ward under continuous supervision by physiotherapists. Future research should consider incorporating additional assessments of pulmonary and exercise function, such as plethysmography, lung diffusing capacity tests (DLCO), and cardiopulmonary exercise testing (CPET).

## 5. Conclusions

Comprehensive rehabilitation after COVID-19 benefits all patients, regardless of the presence of metabolic syndrome. However, individual clinical factors such as age, hypertension, diabetes, and male gender are crucial to its effectiveness. This highlights the need for individualized rehabilitation programs, especially for patients with metabolic conditions, which can significantly increase the effectiveness of therapeutic interventions.

## Figures and Tables

**Table 1:** The characteristics of the groups.

Variable		With MetS *n* (%)	Without MetS *n* (%)	*p*
Sex	Female	40 (47.6)	40 (64.5)	0.063 (0.0052)
	Male	44 (52.4)	22 (35.5)	
Age	Me (Q1–Q3)	67.0 (57.0–74.0)	65.5 (52.0–73.0)	0.257 (0.0214)
Nutritional status (BMI)	M ± SD	31.23 ± 4.73	26.85 ± 4.65	<0.001 * (<0.001)
Hospitalization	Yes	63 (75.0)	39 (62.9)	0.174 (0.0145)
	No	19 (22.6)	21 (33.9)	
Pneumonia during COVID-19 infection	Yes	72 (85.7)	40 (64.5)	0.004 * (0.0003)
	No	10 (11.9)	20 (32.3)	
Oxygen therapy during hospitalization	Yes	58 (69.1)	35 (56.5)	0.450 (0.0375)
	No	18 (21.4)	16 (25.8)	
The duration of rehabilitation	Me (Q1–Q3)	28.0 (21.0–42.0)	23.0 (21.0–38.0)	0.089 (0.0074)
Comorbidities	Diabetes	42 (50.0)	5 (8.06)	<0.001 * (<0.001)
	Hypertension	74 (88.1)	24 (38.7)	<0.001 * (<0.001)
	Asthma	13 (15.5)	3 (4.8)	0.059 (0.0049)
	COPD	3 (3.6)	3 (4.8)	0.699 (0.0557)
Smoking status	Yes	8 (9.5)	4 (6.5)	0.559 (0.0465)
	No	76 (90.5)	58 (93.5)	

Legend: *n*—number of patients; M—mean; SD—standard deviation; BMI—body mass index; COPD—chronic obstructive pulmonary disease; *p*—statistical significance; * *p* < 0.05; ()—result with Bonferroni correction.

**Table 2:** Comparison of lipid profile parameters between patients with and without metabolic syndrome.

Variable		With MetS	Without MetS	*p*
		M (±SD)	M (±SD)	
TC mg/dL	M ± SD	176.0 ± 50.87	181.5 ± 44.59	0.485 (0.097)
HDL mg/dL	Me (Q1–Q3)	35.8 (31.9–45.85)	50.9 (41.6–59.9)	<0.001 * (0.0002)
LDL mg/dL	Me (Q1–Q3)	96.2 (70.6–135.6)	103.7 (74.4–131.3)	0.506 (0.1012)
TG mg/dL	Me (Q1–Q3)	157.5 (127.0–198.5)	110.0 (92.5–131.0)	<0.001 * (0.002)
non-HDL mg/dL	M ± SD	137.6 ± 50.46	129.8 ± 42.28	0.132 (0.0264)

Legend: TC—total cholesterol; HDL—high-density lipoprotein cholesterol; LDL—low-density lipoprotein cholesterol; TG—triglycerides; non-HDL—non-high-density lipoprotein cholesterol; M—mean; SD—standard deviation; *p*—statistical significance; * *p* < 0.05; ()—result with Bonferroni correction.

**Table 3:** The results of multivariate regression analysis for the MetS group.

Outcome	Predictor	OR (95% CI)	*p*
Measurement 1			
FVC	Sex	−10.06 (−21.02, 0.89)	0.071
	Age	−0.02 (−0.53, 0.49)	0.941
	Diabetes	−2.64 (−13.93, 8.65)	0.642
	Hypertension	−3.7 (−24.39, 17.00)	0.722
FEV_1_	Sex	−7.7 (−18.96, 3.56)	0.176
	Age	0.1 (−0.43, 0.62)	0.717
	Diabetes	−6.58 (−18.18, 5.03)	0.261
	Hypertension	−6.91 (−28.18, 14.36)	0.518
6MWT	Sex	32.49 (−21.70, 86.68)	0.235
	Age	−4.2 (−6.73, −1.67)	0.002 *
	Diabetes	−39.46 (−95.33, 16.40)	0.163
	Hypertension	−49.26 (−151.66, 53.14)	0.340
Measurement 2			
FVC	Sex	−10.72 (−20.94, −0.51)	0.040
	Age	−0.06 (−0.53, 0.42)	0.815
	Diabetes	−6.06 (−16.59, 4.47)	0.254
	Hypertension	−7.39 (−26.69, 11.90)	0.447
FEV_1_	Sex	−5.12 (−16.35, 6.12)	0.366
	Age	0.01 (−0.52, 0.53)	0.976
	Diabetes	−7.19 (−18.77, 4.39)	0.219
	Hypertension	−11.72 (−32.95, 9.51)	0.274
6MWT	Sex	45.63 (−23.28, 114.54)	0.190
	Age	−5.34 (−8.56, −2.12)	0.002 *
	Diabetes	−44.84 (−115.88, 26.20)	0.212
	Hypertension	−47.7 (−177.91, 82.51)	0.467
Δ			
FVC	Sex	−0.66 (−7.44, 6.12)	0.846
	Age	−0.04 (−0.35, 0.28)	0.816
	Diabetes	−3.42 (−10.40, 3.57)	0.332
	Hypertension	−3.7 (−16.50, 9.11)	0.566
FEV_1_	Sex	2.58 (−4.36, 9.52)	0.460
	Age	−0.09 (−0.41, 0.24)	0.590
	Diabetes	−0.61 (−7.77, 6.55)	0.866
	Hypertension	−4.8 (−17.92, 8.31)	0.467
6MWT	Sex	13.14 (−38.73, 65.01)	0.614
	Age	−1.14 (−3.56, 1.28)	0.351
	Diabetes	−5.38 (−58.85, 48.10)	0.841
	Hypertension	1.56 (−96.46, 99.58)	0.975

Legend: FVC—forced vital capacity; FEV_1_—forced expiratory volume in 1 s; 6MWT—6 min walk test; OR—odds ratio, CI—confidence interval; Δ—difference between the results after and before rehabilitation; *p*—statistical significance; * *p* < 0.05.

**Table 4:** The results of multivariate regression analysis for the non-MetS group.

Outcome	Predictor	OR (95% CI)	*p*
Measurement 1			
FVC	Sex	−11.04 (−24.93, 2.85)	0.117
	Age	−0.13 (−0.77, 0.51)	0.679
	Diabetes	−21.22 (−46.02, 3.57)	0.092
	Hypertension	16.76 (0.41, 33.11)	0.051
FEV_1_	Sex	−10.45 (−25.08, 4.18)	0.158
	Age	−0.16 (−0.83, 0.52)	0.644
	Diabetes	−10.48 (−36.60, 15.63)	0.424
	Hypertension	15.3 (−1.92, 32.52)	0.080
6MWT	Sex	31.49 (−28.58, 91.55)	0.297
	Age	−5.64 (−8.41, −2.87)	<0.001 *
	Diabetes	−24.13 (−131.34, 83.08)	0.653
	Hypertension	67.05 (−3.63, 137.73)	0.062
Measurement 2			
FVC	Sex	−12.86 (−24.99, −0.72)	0.038 *
	Age	−0.21 (−0.77, 0.35)	0.460
	Diabetes	−25.07 (−46.74, −3.40)	0.024 *
	Hypertension	14.59 (0.31, 28.88)	0.051
FEV_1_	Sex	−12.83 (−26.24, 0.57)	0.060
	Age	−0.03 (−0.64, 0.59)	0.933
	Diabetes	−17.19 (−41.12, 6.74)	0.155
	Hypertension	8.65 (−7.13, 24.42)	0.276
6MWT	Sex	29.78 (−28.25, 87.80)	0.307
	Age	−6.83 (−9.51, −4.16)	<0.001 *
	Diabetes	−51.06 (−154.63, 52.52)	0.327
	Hypertension	0.73 (−67.56, 69.01)	0.983
Δ			
FVC	Sex	−1.82 (−10.88, 7.24)	0.688
	Age	−0.07 (−0.49, 0.34)	0.721
	Diabetes	−3.84 (−20.02, 12.33)	0.635
	Hypertension	−2.17 (−12.83, 8.49)	0.684
FEV_1_	Sex	−2.38 (−9.25, 4.48)	0.489
	Age	0.13 (−0.19, 0.45)	0.414
	Diabetes	−6.71 (−18.97, 5.55)	0.277
	Hypertension	−6.65 (−14.73, 1.43)	0.104
6MWT	Sex	−1.71 (−43.94, 40.53)	0.936
	Age	−1.19 (−3.14, 0.75)	0.224
	Diabetes	−26.93 (−102.32, 48.46)	0.476
	Hypertension	−66.33 (−116.03, −16.63)	0.011 *

Legend: FVC—forced vital capacity; FEV_1_—forced expiratory volume in 1 s; 6MWT—6 min walk test; OR—odds ratio, CI—confidence interval; Δ—difference between the results after and before rehabilitation; *p*—statistical significance; * *p* < 0.05.

## Data Availability

The data that support the findings of this study are available from the corresponding author (A.M.) upon reasonable request.

## Abbreviations

The following abbreviations are used in this manuscript:

6MWT	6-min walk test
FEV1	Forced expiratory volume in the first second
FVC	Forced vital capacity
TC	Total cholesterol
HDL	High-density lipoprotein cholesterol
LDL	Low-density lipoprotein cholesterol
TG	Triglycerides
non-HDL	Non-high-density lipoprotein cholesterol
